# Efficient Adsorption of Chromium Ions from Aqueous Solutions by Plant-Derived Silica

**DOI:** 10.3390/molecules27134171

**Published:** 2022-06-29

**Authors:** Ibeth Guevara-Lora, Norbert Wronski, Anna Bialas, Honorata Osip, Cezary Czosnek

**Affiliations:** 1 Department of Analytical Biochemistry, Faculty of Biochemistry, Biophysics and Biotechnology, Jagiellonian University in Krakow, Gronostajowa 7, 30-387 Krakow, Poland; ibeth.guevara-lora@uj.edu.pl (I.G.-L.); norbert.wronski@doctoral.uj.edu.pl (N.W.); 2 Faculty of Energy and Fuels, AGH University of Science and Technology, Mickiewicza 30, 30-059 Krakow, Poland; anbialas@agh.edu.pl (A.B.); osip@agh.edu.pl (H.O.)

**Keywords:** biomass, biosilica, wastewater, sorption, heavy metals

## Abstract

Nowadays, there is great interest in the use of plant waste to obtain materials for environmental protection. In this study, silica powders were prepared with a simple and low-cost procedure from biomass materials such as horsetail and common reed, as well as wheat and rye straws. The starting biomass materials were leached in a boiling HCl solution. After washing and drying, the samples were incinerated at 700 °C for 1 h in air. The organic components of the samples were burned leaving final white powders. These powders were characterized by powder X-ray diffraction (XRD), Fourier transform infrared (FTIR) spectroscopy, dynamic light scattering (DLS), scanning electron microscopy (SEM), and low-temperature nitrogen sorption. The amorphous powders (biosilica) contained mainly SiO_2_, as indicated by FTIR analysis. Horsetail-derived silica was chosen for testing the removal of dichromate ions from water solutions. This biosilica had a good ability to adsorb Cr(VI) ions, which increased after modification of the powder with the dodecylamine surfactant. It can be concluded that the applied procedure allowed obtaining high purity biosilica from plant waste with good efficiency. The produced biosilica was helpful in removing chromium ions and showed low cytotoxicity to human endothelial cells, suggesting that it can be safely used in environmental remediation.

## 1. Introduction

One of the most serious problems of industrial production is the emission of pollutants that pose a challenge to environmental protection. Among the most dangerous chemical contaminants in wastewater are heavy metal oxyanions, which, even in small doses, can be harmful to the human body [1]. For this reason, finding new cheaper and more environmentally friendly solutions to eliminate these pollutants is a challenge for countries with intensive chemical production. At the same time, new trends regarding the exploitation of natural resources involve the use of waste for the production of adsorptive materials. Agricultural waste has incredible potential benefits, including an effective transformation into materials of high industrial demand, which is economically desirable [2,3]. The use of industrial residues of fruits and vegetables is developing rapidly, while weed waste and crop residues are used less frequently. Since the latter waste can cause serious climate problems, because their accumulation in non-agricultural land is growing rapidly and their fermentation can lead to increased CO_2_ production [4], it would be favorable to find new applications for their use. Furthermore, open burning of plant waste, which is a common practice in many countries, causes the formation of harmful fumes and soot particles that generate smog [5].

Recent publications have summarized the use of biosorbents for the removal of metals from wastewater [6,7]. Several biosorbents derived from different materials, including agricultural and forestry waste, have been tested for metal ion adsorption with high efficiency [8,9]. In this regard, silicon-containing plants have been studied less frequently, although research on the preparation of Si-bearing materials from various sources, with particular emphasis on the use of biomass, is of great interest [10,11,12,13]. For the production of biosilica, mainly rice and wheat husks or straw are used, but other waste, such as sugarcane bagasse or horsetail, are also used [12,14,15,16,17]. It is worth noting that the term biosilica is used to distinguish silica SiO_2_ of inorganic/synthetic origin from silica prepared from biomass. Diverse methods have been applied for the preparation of biosilica, such as thermal techniques, including calcination in reactors or furnaces, and chemical techniques with alkaline extraction or organic acid leaching treatment [12]. The choice of the synthesis method may influence the final product. Generally, the alkaline extraction method can result in the production of high-purity biosilica [14]. However, some disadvantages of these techniques, such as high costs and time consumption, have been reported [12]. In turn, thermal methods are simple and cheap and can be automated but yield products of low purity, which sometimes require additional purification steps. 

In their bibliometric analysis of different methods used to remove heavy metal ions from aqueous environments, Nazaripour et al. have shown that sorption on biosorbents is the most effective approach in terms of environmental impact and cost [18]. A wide range of biosorbents derived from algae, microorganisms, and plant residues have been proposed to remove toxic substances, including heavy metals [3,7]. However, the use of biosilica to remove metal ions from aqueous solutions has been poorly described in the literature [19,20], while the possibilities of using silica produced from synthetic precursors to remove such impurities have been widely reported [21,22,23]. Since SiO_2_ has a relatively large specific surface area (SSA), the use of biosilica as a sorbent seems to be a good solution for the removal of pollutants from the environment. The progressive growth of industrialization causes an increase in the level of environmental pollution, which leads to a growing demand for new materials with good sorption capacities. For this reason, our study focuses on the development of a simple and low-cost method for the preparation of high purity silica powders from a variety of plant waste using a thermal procedure. Moreover, we investigated the usefulness of the biosilica obtained as a sorbent for chromium ions and the level of cytotoxicity of the powders in human endothelial cells. 

## 2. Materials and Methods

### 2.1. Materials

Diphenylcarbazide, dodecylamine (DDA), hydrocortisone, resazurin, and potassium dichromate were obtained from Merck-Sigma (Darmstadt, Germany). Other analytical grade reagents were delivered by POCh (Gliwice, Poland). Endothelial growth factor was supplied by BD Sciences (Franklin Lake, NJ, USA). The cell culture reagents were purchased from Biowest (Nuaillé, France). Cytotoxicity studies were performed with the human dermal microvascular endothelium cell line (HMEC-1) (ATCC, Manassas, VA, USA). Horsetail *(Equisetum arvense)*, common reed *(Phragmites australis)*, wheat straw *(Triticum aestivum)*, and rye straw *(Secale cereale)* were used for the preparation of silica. Biomass samples were collected from a local farm located in south-eastern Poland. 

### 2.2. Preparation of Silica Samples

**Figure 1:**
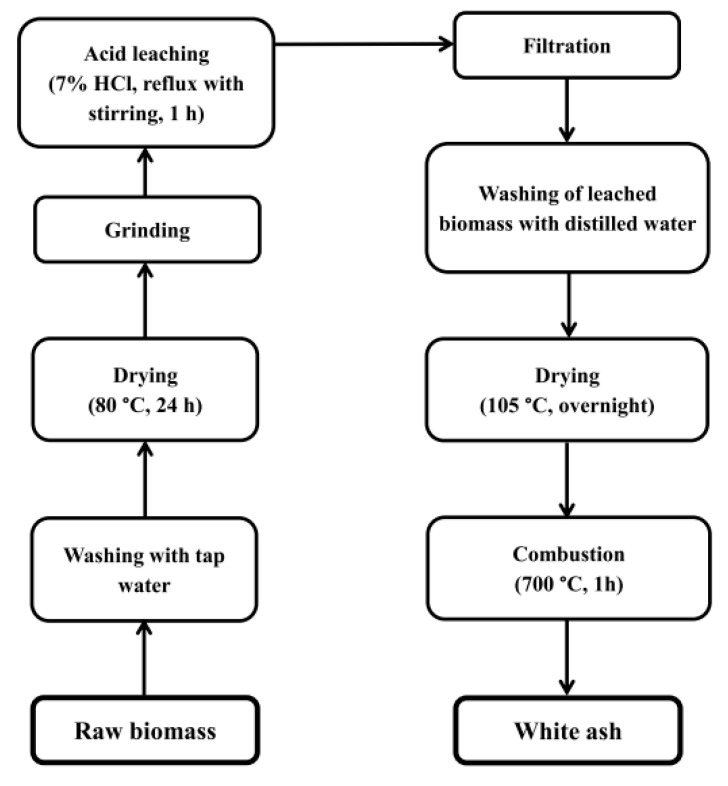
Scheme of the two-step process for the preparation of silica powders from various types of biomass.

Silica powders were produced from plant biomass using a simple two-step process (Figure 1). All plants were washed with water to remove soluble contaminants and solid particles such as dust or sand. The biomass was then dried in an air oven at 80 °C for 24 h. The dried plants were cut into approximately 1 cm long pieces, ground, and sieved through a 1-mm round mesh screen. The powdered samples were heated in a round bottom flask equipped with a magnetic stir bar and refluxed in a 7% HCl solution for 60 min to remove metallic impurities. The mixture was then filtered on a Büchner funnel under partial vacuum. The precipitate was washed several times with distilled water and left to dry in an oven at 105 °C overnight. The dried samples were incinerated in an electrical muffle furnace at 700 °C with atmospheric air flow for 60 min. The obtained white silica powders were stored in a desiccator for further analysis.

### 2.3. Modification of Silica Powder with Dodecylamine

The horsetail-derived silica powder was modified with DDA [24]. Briefly, 50 mL of a 6 M HCl solution were added to 1 g of the powder and incubated at 60 °C for 2 h with constant agitation (150 rpm) in the Orbital Shaker-Incubator ES20/60 (Biosan, Riga, Latvia). The mixture was incubated overnight at room temperature. After removing of HCl solution, the powder was thoroughly rinsed with distilled water, and 100 mL of a 1% DDA solution were added. The sample was mixed continuously for 1 h at 60 °C (150 rpm). After incubation, the powder was rinsed with distilled water and dried at 60 °C. 

### 2.4. Characterization of Powders

The mean particle size of the powders was estimated using DLS technique with the Zetasizer Nano S device (Malvern Panalytical, Malvern, UK). The morphology of the selected powders was examined by SEM (Hitachi, Tokyo, Japan, model S-4700). The textural properties of the materials were tested by nitrogen sorption at –195.8 °C using 99.999% purity nitrogen as an adsorptive with the use of a Gemini V 2380 analyzer (Micromeritics, Norcross, GA, USA). Before measurement, the samples were degassed at 350 °C under dynamic vacuum for 2 h. The specific surface area (SSA) was determined using the Brunauer–Emmett–Teller (BET) method. The pore size distribution was evaluated with the Barrer–Joyner–Halenda (BJH) model from the desorption branch. XRD analysis of the powders was performed with an Empyrean diffractometer (PANalytical, Malvern, UK) with a Cu Kα source. All powders were characterized by FTIR spectroscopy (Nicolet 380, Thermo Electron Corp., Waltham, MA, USA) using KBr pellets containing approximately 1 mg of sample. The spectra were obtained in the range of 4000 to 400 cm^−1^.

### 2.5. Adsorption Study

The adsorption of chromium ions on biosilica was investigated using potassium dichromate solutions. The concentration of Cr(VI) ions in the solutions was determined by a colorimetric test with diphenylcarbazide [25]. SiO_2_ powders (5 mg) were incubated with 250 μL of dichromate solutions at various concentrations (2.5–50 μg/mL) for 60 min at 25 °C with constant agitation (150 rpm) in Orbital Shaker-Incubator ES20/60 (Biosan, Riga, Latvia). After incubation, the samples were centrifuged (5 min, 10,000 rpm) and 50 μL of supernatant were added to 0.930 mL of 0.05 M sulfuric acid in a spectrophotometric cuvette. Next, 20 μL of a 0.25% diphenylcarbazide solution in acetone were added and carefully mixed. After 15 min of incubation, the absorbance was measured at λ = 546 nm. The initial concentration of the metal ions in each solution was also measured in the same way. In each experiment, a standard curve was created for the measurement of dichromate ions (0–50 μg/mL), achieving a high reproducibility. Similar experiments were carried out with DDA-modified biosilica. 

The amount of adsorbed dichromate ions was calculated from the difference between the initial and final concentrations of the ions in the solution used for the adsorption tests and normalized to 1 mg of biosilica powder according to the equation:

$$q=\frac{\left(c_{0}-c_{f}\right)V}{m},$$

where *c*_0_ (mg/L) is the initial concentration of metal ions, *c_f_* (mg/L) is the concentration of metal ions in the solution after incubation with biosilica powder, *V* (L) is the volume of the metal ion solution used for the sorption test, and *m* (g) is the weight of the powder.

### 2.6. Cytotoxicity of Biosilica Powders

Human microvascular endothelial cells (HMEC-1) were cultured in Endothelial Basal Medium supplemented with an antibiotic/antimycotic solution (100 U/mL penicillin, 0.1 mg/mL streptomycin, and 2.5 μg/mL amphotericin B) and with 5% fetal bovine serum. Cell growth was induced by the addition of hydrocortisone and endothelial growth factor at a final concentration of 10 μg/mL and 10 ng/mL, respectively. In the cytotoxicity experiments, cells were cultured without growth factors. The effect of biosilica on cell viability was evaluated using the Alamar Blue assay. Cells (6 × 10^3^) seeded in a 96-well microplate previously precoated with 0.01% gelatin were treated with biosilica particles suspended in cell medium in a concentration range of 0.0125 to 1 mg/mL for 48 h. Next, after removing the medium and washing the cells, 100 µL of a 0.001% resazurin solution were added to each well. The cells were then incubated for four hours at 37 °C. After that time, the supernatant fluorescence was measured using a Synergy H1 microplate reader (BioTek Instruments, Winooski, VT, USA) at λ_ex_ = 560 nm and λ_ex_ = 590 nm. Simultaneously, cell viability without biosilica (control sample) was also measured.

### 2.7. Statistical Analysis

The experimental results of dichromate ion sorption on biosilica and cell viability were fitted using GraphPad Prism 8.0.1 software (GraphPad Software, San Diego, CA, USA).

## 3. Results and Discussion

In this study, high purity silica powders were obtained from raw biomass, i.e., horsetail, reed, and wheat and rye straws, using a low-cost two-step acid pretreatment/incineration process. The silica preparation yield from horsetail was approximately 20 g per 100 g of biomass, while the other sources provided 4–6 g of silica per 100 g of biomass. The silicon content in the dry matter of raw horsetail has been reported to be in the range of 20–30 g/kg [26]. Therefore, it can be stated that the efficiency of the method used in this study for the preparation of SiO_2_ is significantly high. This may be related to the use of hydrochloric acid, which removes metallic impurities from biomass and can also eliminate some organic components of the starting material [15]. It is worth mentioning that the silicon content in plants depends on the fertilization used and the properties of the soil [27,28]. Guerriero et al. also demonstrated that the amount of silica in silicon-containing plants may be related to the concentration of certain polysaccharides in the plant, such as callose [29]. 

The sizes of the powders obtained are in the nanometer and micrometer ranges, as determined by DLS. The horsetail-derived silica had the finest particles. The average particle size ranged from 166 to 890 nm (Table 1). In turn, thicker particles with sizes in the range of 1867 to 5521 nm were obtained from the common reed. The straw-derived particles were medium-sized. These values are in agreement with those found in other reports [30,31]. 

**Figure 2:**
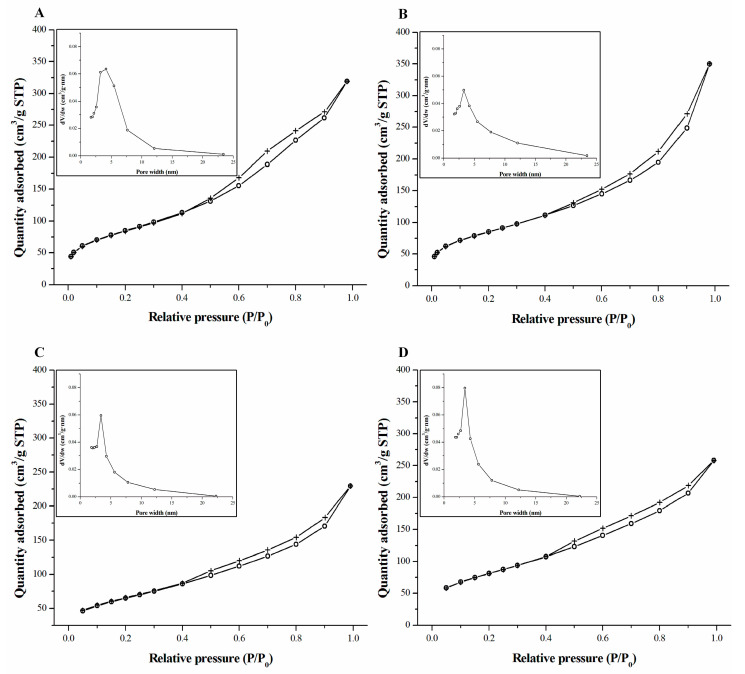
Nitrogen adsorption-desorption isotherms for silica powders obtained from: (**A**) horsetail, (**B**) common reed, (**C**) rye straw, and (**D**) wheat straw. The adsorption and desorption curves are presented with open circles and crosses, respectively. The insets show the pore size distribution of the studied powders.

The results of nitrogen sorption on biomass-derived silica powders are presented in Figure 2. According to the IUPAC classification, the presented isotherms can be classified as mixed type II and type IV. A characteristic feature of these curves is the presence of a hysteresis loop usually attributed to capillary condensation of the adsorbate in mesoporous structures [32,33]. The shape of the hysteresis loops may be classified as type H3, which corresponds to the presence of non-rigid aggregates of plate-like particles causing the formation of slit-shaped pores [33]. BJH calculations indicated a narrow pore size distribution with maximum values of about 4 nm for all silica powders, which are close to those reported in the literature [14,17] (Figure 2A–D, insets). Nevertheless, the pore size of biosilica depends on the material from which it is produced. For example, biosilica from different types of rice husks had different pore diameters ranging from 5 to 35 nm [34].

The SSA values (Table 2) obtained for all the silica powders produced in the present study were comparable and did not differ significantly from those obtained with a similar procedure from wheat straw or horsetail [15,17].

Previous reports on silica preparation from sugarcane bagasse and rice husks using chemical techniques showed SSA values lower than those observed in this study, suggesting an impact of the preparation technique on the textural properties [14,34]. It should be noted that the ash samples obtained in this study directly from the incineration of the studied biomass without previous HCl treatment exhibited SSA values of approximately 1 m^2^/g. Such low values may be related to the relatively high potassium content in this biomass [35]. At elevated temperatures, potassium can react with Cl, P, S, and Si present in biomass to form potassium-containing compounds with a low melting point or eutectics [35,36,37]. The molten phases formed during biomass combustion can favor the sintering of ash particles but are also likely to fill open pores, leading to a reduction in SSA. Biomass pretreatment with an acid solution allows the removal of potassium compounds and other metallic components what can prevent the sintering of silica particles during combustion. 

**Figure 3:**
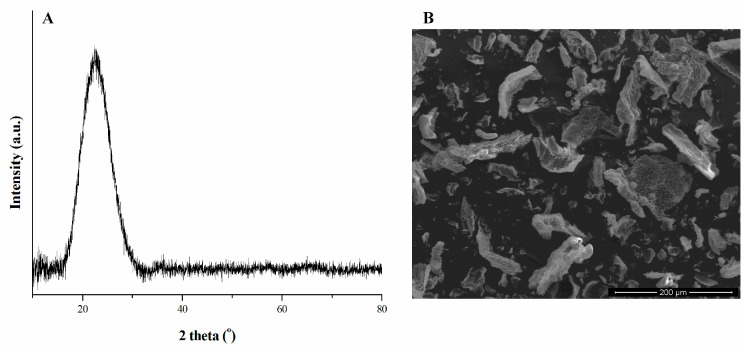
(**A**) XRD pattern of horsetail-derived silica powder, (**B**) SEM image of the powder, magnification 350×.

The XRD pattern of the biosilica powder obtained from horsetail is presented in Figure 3A. The only visible feature is a wide diffraction band (2θ in the range 15°–30°), which indicates the amorphous nature of the powder. The typical SEM image of this silica material shows that the original surface morphology of the initial sample is preserved both during acid treatment and during combustion (Figure 3B). The irregular particle shapes resulted from the comminution of the starting material. These morphological and physicochemical properties are similar to those observed for biosilica powders obtained with similar preparation methods [17,30].

**Figure 4:**
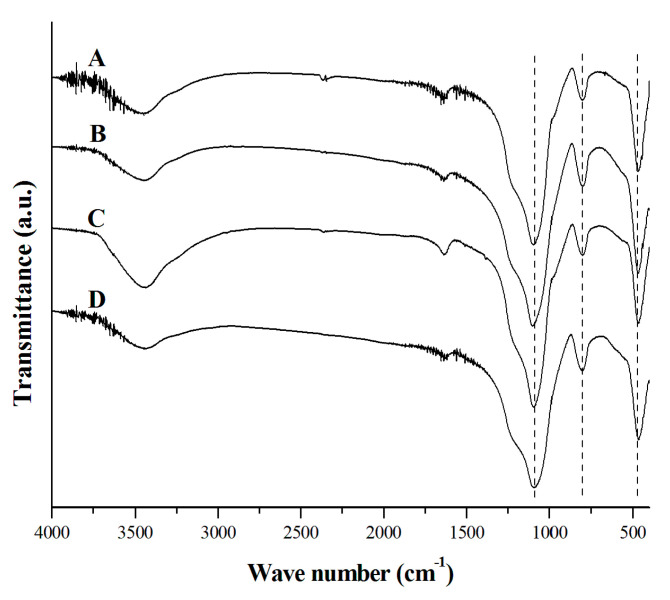
FTIR spectra of silica powders. (A) horsetail, (B) common reed, (C) rye straw, (D) wheat straw. The characteristic bands for SiO_2_ are marked with dashed lines.

FTIR analysis was performed to determine the qualitative composition of the materials studied (Figure 4). Regardless of the origin of the biosilica samples, their spectra contain absorption bands that correspond to the same functional groups. The broad band at ca. 3430 cm^−1^ and the band at ca. 1630 cm^−1^ are attributed, respectively, to the symmetric/asymmetric stretching O–H and O–H bending modes of the Si–OH groups and/or adsorbed water [17]. Silanol groups often appear on the surface of silica and are responsible for the adsorption of water molecules [38]. There are three strong bands at 1088, 803, and 470 cm^−1^, which can be assigned to Si–O–Si asymmetric stretching, symmetric stretching, and bending modes of silica, respectively [17]. This confirms that silica SiO_2_ is the main component of powders prepared from all types of biomass.

**Figure 5:**
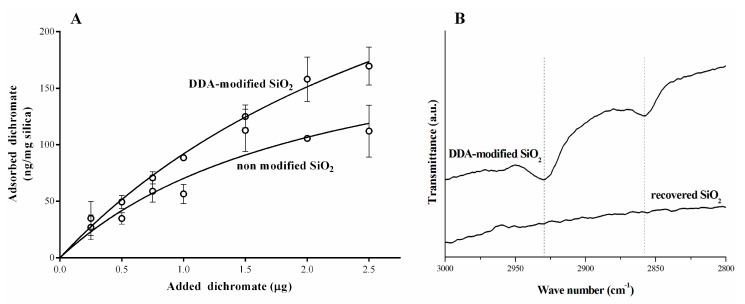
(**A**) Plot of dichromate adsorption on horsetail-derived silica powders. The amount of dichromate ions bound to silica particles is presented as a function of the total amount of the added dichromate ions. The data points present the mean ± SD of three independent experiments. Wilcoxon matched-pairs signed rank test was used to establish the statistical difference between either curves; *p* < 0.05. (**B**) FTIR spectra of horsetail-derived silica powder modified by DDA and the powder after the recovery process.

Since the horsetail-derived silica powder was obtained more efficiently and the physicochemical properties of all powders did not differ significantly, further studies on biosilica applicability for Cr(VI) ion sorption were conducted using this powder. Dichromate anion (Cr_2_O_7_^2−^), i.e., one of the most common pollutants, is known to be carcinogenic and mutagenic in nature [39]. For this reason, the removal of Cr(VI) from wastewater is an essential environmental goal. A typical adsorption curve showing the dependence of the amount of dichromate ions adsorbed on 1 mg of powder as a function of the total amount of added dichromate ions is presented in Figure 5A. The theoretical value of the maximum adsorption capacity per mg of biosilica powder (B_max_) was calculated after mathematical fitting to the experimental points using Langmuir model. The calculated B_max_ for the adsorption of dichromate ions in horsetail-derived silica reached a value of 220 ng per mg of powder. This value is relatively lower than the higher values for maximum adsorption capacity reported in other studies [22,40,41]. It should be noted that these reports concerned nanosilica products obtained by chemical synthesis without biological sources, while the powder used in this study was derived from biomass and contained submicrosized grains that cannot be classified as nanomaterial. Moreover, in the studies mentioned above, the nanosilica surface was modified by introducing functional groups, leading to improved sorption. For this reason, we tried to modify biosilica in order to improve its adsorption capacity. An easy, economical, and reversible modification with DDA surfactant was applied.

Different chemical modifications of synthetic adsorbents have been proposed to improve their adsorptive properties [19,42]. Mesoporous silica is believed to be a good material for functionalization because of its large surface area, narrow pore size distribution, and controlled pore size. DDA surfactant is frequently used to modify the surface of adsorbents [19,24]. It is an aliphatic hydrocarbon having an amino group. The introduction of neutral and protonated amino groups into silica can result in better adsorption of cations or anions, respectively [19]. Biosilica materials modified in this way can also remove heavy metals and their oxyanions by ion exchange mechanism. In this study, the horsetail-derived mesoporous silica was modified with DDA and analyzed using the FTIR technique to confirm the presence of characteristic groups of the surfactant (Figure 5B).

In fact, the appearance of additional bands (2858 cm^−1^ and 2929 cm^−1^) can be assigned to the representative groups of DDA: asymmetric stretching vibrations (−CH_2_) and symmetric (−CH_2_) vibrations of the methylene groups, respectively [43]. The modification of horsetail-derived silica with DDA showed a typical adsorption curve, and the calculated B_max_ increased twofold compared to the non-modified silica—425 ng of Cr(VI) per mg of powder (Figure 5A). The maximum acceptable concentration of dichromate ions in municipal and industrial wastewater is 0.05 mg/L or 0.1 mg/L, respectively [44]. Our results showed that the DDA modified biosilica was able to adsorb a maximum of 425 µg of dichromate ions per g of powder confirming its effectiveness in removing chromium ions. The maximum quantity of ions that can be adsorbed per 1 g of modified silica is eight times higher than the maximum acceptable amount of dichromate per liter of wastewater. In addition, spent powders resulting from the adsorption of dichromate ions can be recycled by treatment with weak inorganic acids. Such attempts were made, and the recovered silica showed a high degree of purity, as determined by FTIR (Figure 5B).

**Figure 6:**
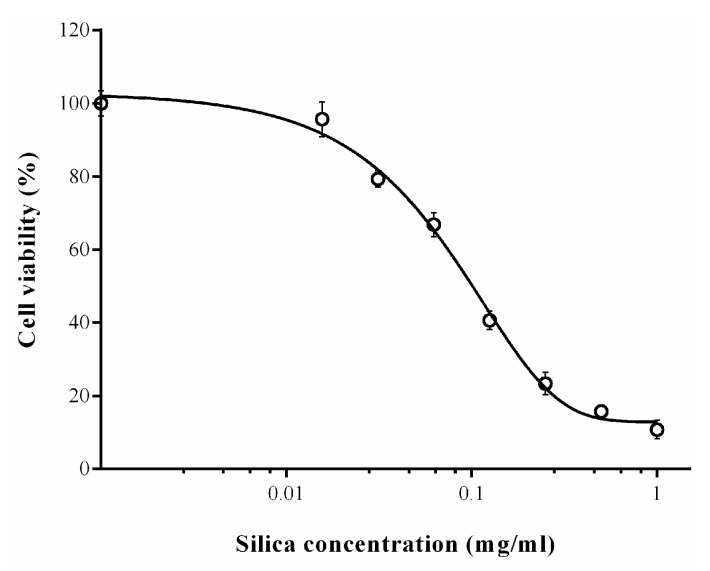
Cell viability after horsetail-derived silica treatment. The cytotoxicity of the prepared powder is presented as the percentage of viability of treated cells compared to untreated cells, whose viability was assumed to be 100%. The data points present the mean ± SD of three independent experiments.

Several studies have been conducted to elucidate the adverse effects of amorphous silica, especially as nanoparticles [45]. SiO_2_ nanoparticles have been shown to have toxicity against many human cells through mechanisms related to an increased inflammatory response, oxidative stress leading to cell necrosis or apoptosis [46]. In this regard, the cytotoxicity of horsetail-derived silica was analyzed in human endothelial cells (Figure 6). The results were presented as the percentage of living cells after treatment compared to the viability of untreated cells, which was assumed to be 100%. The tolerance of these cells to biosilica powder is not significantly different from that observed in previous studies, which showed that the cytotoxicity of silica was dependent on the size of the particles: the smaller the size, the higher the toxicity [47,48]. In these reports, silica powders with a size greater than 150 nm revealed less toxicity than nanosilica (<100 nm), showing a minimal dose tolerance of less than 50 µg/mL. In this study, a similar value (65 µg/mL) was obtained. Therefore, we can assume that the powders obtained by us can be used safely, probably due to their particle size, which varies from 160 to 850 nm. Moreover, Zhao et al. showed that modified nanosilica was less toxic to endothelial cells than unmodified [49]. This report showed that the introduction of amino and carboxyl groups on 15-nm silica grains resulted in a significant reduction in toxicity. Therefore, we can assume that the DDA-modified biosilica may show lower cytotoxicity. These issues require more in-depth research and should be addressed in a variety of cell types, especially immune cells and skin cells. 

## 4. Conclusions

This paper presents an attempt to develop a cheap and efficient preparation of high-purity amorphous biosilica powders from silicon-bearing plants. The advantage of the proposed solution is the abundance of plant-based biomaterials without the need for cultivation of dedicated crops. This allows to prepare biosilica with high efficiency at low costs. FTIR results showed high purity of the SiO_2_ powders obtained from each type of biomass. Although the final powders had different grain sizes, they showed a relatively high SSA value of the same order or even higher than that reported in the literature on silica obtained from other sources. This feature determined the use of these products to test their suitability for the removal of highly toxic chromium ions from aqueous solutions. Such a study was carried out on horsetail-derived biosilica. The results demonstrated effective adsorption of dichromate ions, which was higher when the biosilica was initially modified with the DDA surfactant. This finding suggests that the biosilica tested can be successfully used to remove various toxic pollutants from wastewater. Moreover, it should be noted that the amount of adsorbed dichromate ions per gram of biosilica was eight times higher than the maximum acceptable content of ions in wastewater. Due to the low cytotoxicity of biosilica in relation to endothelial cells, it can be assumed that its use in sewage treatment plants will not generate an additional risk for the biosystem. In conclusion, the technique used in this study provides a useful way of utilizing waste to obtain valuable biomaterials with high applicability, such as the removal of pollutants. 

## Figures and Tables

**Table 1:** Average size of silica powder particles derived from different types of waste biomass.

Biomass	Mean Size(nm)
Horsetail (*Equisetum arvense)*	166 ± 34 688 ± 49 890 ± 93
Common reed (*Phragmites australis)*	1867 ± 668 5521 ± 55
Wheat straw (*Triticum aestivum*)	422 ± 38 1395 ± 245
Rye straw (*Secale cereal*e)	317 ± 6 1199 ± 180

**Table 2:** Specific surface area (SSA) of silica powders prepared from different types of waste biomass. SSA was determined using the BET method on the basis of low-temperature adsorption isotherms of nitrogen.

Silica Source	Horsetail	Common Reed	Wheat Straw	Rye Straw
SSA (m^2^/g)	305	249	293	236

## Data Availability

The data presented in this study are available on request from the corresponding author.

## Sample Availability

Samples of the biosilica are available from corresponding author upon reasonable request.
